# Comparative Quality Control Study of Different Brands of Diclofenac Sodium Tablet Available in Local and Government Pharmacies by In-Vitro Testing

**DOI:** 10.7759/cureus.11348

**Published:** 2020-11-05

**Authors:** Madan Mohan Gupta, Avinash Khoorban, Ahamad Ali, Ornella Ramlogan, Debjyoti Talukdar

**Affiliations:** 1 School of Pharmacy, Faculty of Medical Sciences, The University of the West Indies, St. Augustine, TTO; 2 College of Pharmacy, Teerthanker Mahaveer College of Pharmacy, Moradabad, IND

**Keywords:** diclofenac sodium, quality control testing, weight variation, disintegration, friability, drug assay, dissolution

## Abstract

The objective of this study is to conduct in-vitro quality control testing of diclofenac sodium tablets involves weight variation test, drug assay, friability test, and the disintegration and dissolution test. Two brands of diclofenac sodium tablets were used in the study, named Brand A and Brand B. Quality control (QC) test results for diclofenac sodium tablets show that both Brand A and Brand B conform to the United States Pharmacopeia (USP) standards. In terms of weight variation, Brand A and B have an above the mean weight limit variation of 2.79% and 2.05%, respectively. The lower mean weight limit variations are 1.21% and 1.27%, respectively, which are within the 10% standard limits of USP. Friability tests show that Brands A and B have an average friability of 0.062% and 0.01% mass loss, which are within the 1% mass loss limits of USP. In terms of drug assay, both Brands A and B fall under the USP parameter of 85%-115%, respectively. The disintegration test shows that Brand A and Brand B fall within a 15-minute time interval segment with disintegration time calculated as 6.69 min and 7.02 min for Brands A and B, respectively. Brand B of Diclofenac Sodium has a drug dissolution percentage of 90.7% within a 45-min sampling time interval.

Brands A and B pass the pharmacopeia limits set under the USP standards. The friability test shows that the loss of mass for both Brands A and B was within the 1% standard limit. Similarly, with regard to weight variation, both brands conform under the normal limit of 10% above or lower the mean weight. In terms of drug assay, both brands' drug availability was within the specified 85%-115% standard range. They passed the disintegration and dissolution test within a time limit of less than 15 minutes and 45 minutes, respectively.

## Introduction

Diclofenac sodium is a non-steroidal anti-inflammatory drug (NSAID), which is used for inflammation, joint stiffness, and rheumatic and non-rheumatic conditions. It has a potentially short half-life (approx 2 hours) and the drug can be administered orally, rectally, or intramuscularly. It is comparable to other analgesics, has a fast duration of action, and limits potential drug accumulation in the body [1]. The study is intended to analyze certain critical parameters in terms of quality control testing of the diclofenac sodium tablets marketed by various manufactures using an in vitro test. The quality testing involves a wide range of instruments such as friabilator, dissolution test apparatus, disintegration test apparatus, ultraviolet-visible spectrophotometer, mixer, digital pH meter, electronic weighing balance, etc. Some of the tests performed are as follows: friability, disintegration test, weight variation test, drug assay, dissolution test, drug content uniformity, etc. [2]. The study's aim was to check the quality parameters of the marketed diclofenac tablet by in vitro testing to ensure that all tablets possess the pharmacopoeial limits.

Diclofenac sodium tablets are generally available over-the-counter (OTC) and prescribed as NSAIDs worldwide, along with other drugs. It is important to make sure that various brands of diclofenac sodium adhere to the quality criteria guidelines as required. The studies show that diclofenac sodium inhibits cyclooxygenase (COX) [3]. It also inhibits N-methyl-D-aspartate (NMDA) receptor, substrate P, and peroxisome proliferator-activated receptor gamma (PPAR-γ or PPARG). It leads to reduced pain perception or the transduction of neuropathic pain. Diclofenac sodium tablets are associated with anti-inflammatory and analgesics, as they inhibit COX1, COX2, prostaglandin, prostacyclin, and thromboxane [4]. Prostaglandin is produced during the inflammatory process and its inhibition aids in reducing the inflammation.

Pharmaceutical characteristics of sodium diclofenac tablet

Tablets are coated and compressed in a variety of strengths. Some tablets, like effervescent, are dissolved in water before administration, and there is a sublingual release of the drug through the sublingual route. Sodium diclofenac is effective and efficacious as solid dosage form tablets. The purpose of the research is to focus on the effective and efficacious aspects of the tablet in the type of pharmaceutical dosage form. The pharmaceutical ingredients of the tablet include active pharmaceutical ingredients and excipients [5]. Incipients involve active pharmaceutical ingredients while excipients may include ingredients that promote efficient tableting, disintegrants, binders, diluent, lubricants, etc. Polymer coating around the tablet aids in extending the drug shelf life and appearance as well.

Diclofenac sodium tablets are available in various sizes and shapes. The ingredients of the tablet play an important in terms of the formulation of the tablet. Particle size, uniformity, and active ingredients also play a crucial role. Binders like lactose, cellulose, and hydroxypropyl methylcellulose (HPMC) also play a crucial role, as they bind the tablet and disintegrate it when it comes in contact with the digestive tract [6]. The study tested certain features like friability in order to ensure that the tablet strength is good and during transportation, the loss of tablet will be in the pharmacopoeial limit; this is also related to the hardness of the tablet. The tablet should be able to resist certain stresses, such as commercial packaging, shipping, and handling, which can lead to a deformity in its physical form, [7]. The study is quite important, as it forms the basis to check the quality of a tablet on a commercial scale through quality control (QC) testing.

QC testing

Quality control (QC) testing ensures drug safety, efficacy, and effectiveness. It involves specific instruments to ensure the quality of drug testing as per set guidelines. Some of the testing procedures are as follows: friability, weight variation test, disintegration test, dissolution test, and drug assay. The equipment used are as follows: friabilator, electronic weighing balance, mixer, ultraviolet (UV)-visible spectrophotometer, digital pH meter, dissolution test apparatus, and disintegration test apparatus [7-8]. Friability tests the content uniformity and weight variation. It involves the tendency of the tablet to fragment, powder, or chip, which could affect the appearance and uniformity of the drug, while weight variation involves drug distribution uniformity. The weight variation test is applicable when the drug substance is more than 50 mg or 50% by weight of the tablet. The disintegration test involves the time required to break tablet components into particles. It also involves a 10-mesh screen and time is measured while disintegrated particles pass through the mesh screen. The bioavailability of the drug is measured through a dissolution test. It refers to the amount of drug that goes into a solution as per unit time under standardized conditions [8]. The drug assay investigates in vitro quality control testing. It measures and determines the quantity and quality of the specified analyte using the UV-visible spectrophotometer through the amount of radiation absorbed. It also includes the Beer-Lambert law, which relates to the attenuation of light and the properties of the material through which it is passing.

Overall, it is important to maintain the quality of a product by performing various quality control (QC) testing upon it to make sure that it is safe for the public domain. At the same time, it maintains the efficiency and quality of the overall product. QC testing also ensures that the drug adheres to the details as per the description and data stated on the drug label. It involves checking the purities and impurities in a drug, active ingredients, drug absorption by the body, etc. The in vitro testing performed in this research determines the quality, efficacy, and effectiveness of diclofenac sodium drug as per United States Pharmacopeia (USP) and British Pharmacopeia.

Characteristics of diclofenac sodium

As per the drug profile, diclofenac sodium belongs to the class of NSAIDs class, which is solid at room temperature. It appears as white colored crystals and possesses both analgesic and antipyretic properties. It is prescribed to treat pain, ankylosing spondylitis, actinic keratosis, osteoarthritis, ocular inflammation, etc. The mode of action depends upon leukocyte migration inhibition, cyclooxygenase (COX1 & COX2) inhibition, and prostaglandin synthesis inhibition [9]. It also dissipates heat, causing peripheral dilation with increased cutaneous blood flow. In terms of absorption, 50% of the drug reaches the systemic circulation as it undergoes first-pass metabolism. The time taken to reach the maximum concentration is 5.3 hours. The volume distribution (V/F) is 1.4 L/kg. The drug is primarily bound to albumin (99%) and the biological half-life of the drug is one to two hours [10]. The dissociation constant pKa of the drug is 4.15 and water solubility is 2.37 mg/L at 25ºC.

## Materials and methods

QC testing of the diclofenac sodium drug involves the following equipment: electronic weighing balance (Adam AFA-120LC, Adam Equipment, Milton Keynes, UK), UV visible spectrophotometer (Agilent 8453; Agilent Technologies, Santa Clara, California), mixer (Maxi Mix II), digital pH meter (OAKTON Instruments, Vernon Hills, IL), friabilator (Electrolab EF2, Electrolab India Pvt. Ltd., Mumbai, India), dissolution test apparatus (Electrolab EDT O8Lx; Electrolab India Pvt. Ltd.), and disintegration test apparatus (Electrolab ED 2L; Electrolab India Pvt. Ltd.). Some of the materials used in the in-vitro QC test are as follows: diclofenac sodium pure drug (Sigma-Aldrich, St. Louis, Missouri), commonly available diclofenac sodium tablets (Brand A and Brand B) conventional, Tulsion-412 (cholestyramine resins manufactured by Cadila Pharmaceuticals, Ahmedabad, India), sodium hydroxide, potassium dihydrogen phosphate, and distilled water (pharmaceutical grade). The tests performed with the equipment mentioned above are as follows: friability, disintegration test, weight variation test, drug assay, and dissolution test.

Weight variation test

This test examines uniformity in accordance with the formulation of each batch of tablets, which illustrates its content. In this study, we selected 20 tablets of diclofenac sodium from Brand A, which were weighed individually and collectively. Weight variation was calculated using the formula - (Initial weight - Average weight)/Average weight X 100. It is meant to compare the USP limits and the data were recorded in table format [11]. The same procedure was repeated for Brand B.

Drug assay

A drug assay is crucial, as it validates the drug as per the labeled amount. It checks the dosage formulation as per the desired formulation based upon precision, accuracy, robustness, and selectivity. In this study, 20 50 mg diclofenac sodium tablets were randomly selected and crushed into a powder. Fifty ml of distilled water was added to 53.05 mg of the crushed powdered drug, which corresponds to 10 mg of active diclofenac sodium drug. Sonification was performed to agitate the particles in the mixer using sound energy. The process was performed using the instrument Maxi Mix II, which also maintained the uniformity of the mixture [11]. The contents were made up and divided into 100 ml using a volumetric flask and 1 ml of the solution was pipetted and diluted to 10 ml, which was placed in the UV visible spectrophotometer. The wavelength of absorbance of the spectrophotometer was set to 276 nm max. The calibration curve was utilized to illustrate the available amount of drugs in both brands.

The UV spectrophotometer is useful in terms of quantitative analysis, as certain organic compounds can be identified using it. Apart from that, it helps measure the function of ratio using the intensity of the two beams of lights in the UV visible range, as it measures the amount of UV or visible radiation absorbed by the substance in the solution.

Friability test

As per USP standards, the friability test was performed using the Roche friabilator [6]. It checks the tendency of the tablet to crumble, chip, or break upon abrasion or compression. It is important to check the friability of a tablet for complete dissolution in the gastrointestinal tract. The test checks the sturdiness of a tablet, and a loss of 1% tablet mass is acceptable during the process.

Disintegration test

As per USP standards, the diclofenac sodium tablets are disintegrated into small granules to increase the surface area [6]. It involves the disintegration of the tablets in a liquid medium as stated in the monograph under experimental conditions and recorded as disintegration time. The test is crucial, as it provides critical safety data in regard to the bioavailability of the solid dosage form of the drug. During this test, six tablets from Brand A were randomly selected and one tablet was placed in each test tube with a mesh size of 10 basket as per USP standards. The basket was placed in a 1 liter beaker containing phosphate buffer solution of pH 6.8 at 37°C. The apparatus was stirred at 28-32 cycles/minutes and, simultaneously, a stopwatch was started. When all the particles passed from each test tube into the beaker, the finishing time was noted as the disintegration time. The same procedure was repeated for Brand B. This disintegration test was a quantitative test, as time was measured during the test.

Dissolution test

The dissolution test is crucial to ensure the therapeutic effectiveness of a drug and its bioavailability. It measures the extent of solution formation. In this test, we required a 10L phosphate buffer at pH 6.8. The following formula was used as a basis for preparing the buffer - 6.8 g KH2PO4 + 0.94 g NaOH dissolved in 1L of distilled water gives a phosphate buffer of pH 6.8. In order to prepare a 10L buffer solution, the following ingredients were used - 68 g of KH2PO4 and 9.4g of NaOH were dissolved in 0.5L distilled water in a 1L volumetric flask. The flask was shaken to form a solution. The same procedure was repeated to expand the solution to make a 10L buffer using distilled water. In order to attain 6.8 pH for the buffer, HCl and NaOH were used accordingly to adjust the pH.

The following equipment was used for the dissolution test - Electrolab EDT (Electrolab India Pvt. Ltd.) - 08Lx Type 2 [6]. A type 2 dissolution instrument was used because as per the monograph of the tablet, a paddle (Type 2) instrument is used for the dissolution study of the tablet. This test provides us a correlation between in vitro and in vivo along with the efficacy of the dosage form. The dissolution beaker was filled with 900 ml of phosphate buffer at 6.8 pH and heated to 37±0.5°C. One tablet from Brand A was added to each beaker and stirred at 75 rpm. At specific time intervals - 5, 10, 15, 30, 45, 60, and 75 min, respectively, 5 ml of the solution was removed and 5 ml of the buffer solution was added. The absorbance of diclofenac sodium was determined using UV visible spectrophotometer at the above-mentioned time intervals.

## Results

The QC tests performed are as follows: weight variation, drug assay, friability, disintegration, and dissolution tests. All tests performed were within the pharmacopeia limits. It is crucial that commonly prescribed NSAIDs like diclofenac sodium conform as per USP standards in order to maintain drug safety, efficacy, and effectiveness in the human body.

Weight variation test results

**Figure 1:**
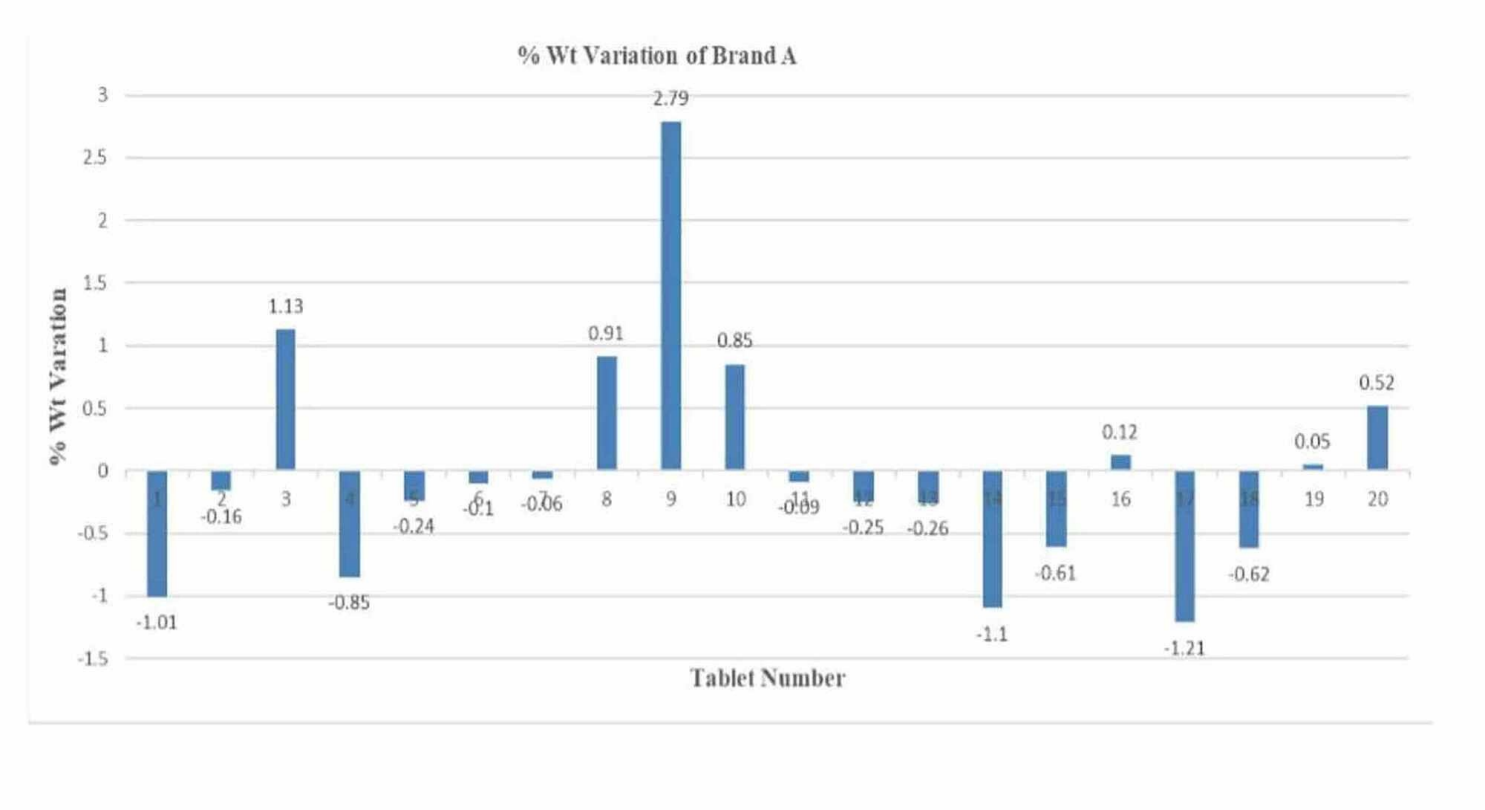
Graph of percentage weight variation for Brand A

**Figure 2:**
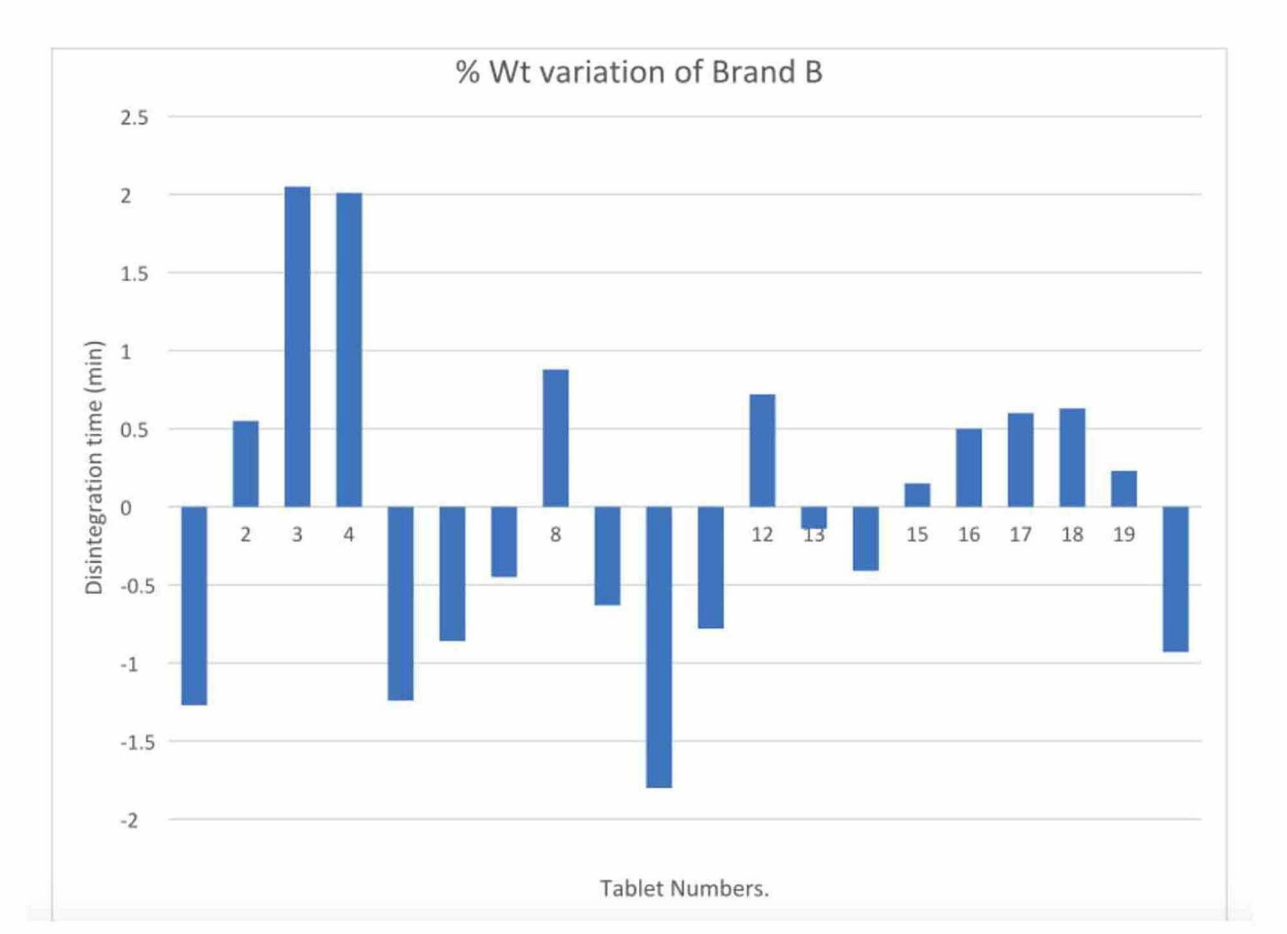
Graph of percentage weight variation for Brand B

The weight variation test results are meant to maintain drug uniformity and distribution. The test results show that both brands of diclofenac sodium - Brand A and Brand B - conform to USP standards [6]. The highest percentage variation for Brand A above the mean was 2.79% and the lowest percentage variation below the mean was 1.21%. There were a total of 20 tablet readings for each brand. Each tablet was weighed three times. The average of all three readings was calculated, rounded off, and the percentage weight variation was calculated as mentioned in the procedure above. A graph with the percentage weight was plotted for both Brand A and Brand B. For Brand B, the highest weight variation above the mean was 2.05% and the lowest weight variation below the mean was 1.27%. The results are given in Tables 1-2 and Figures 1-2.

**Table 1:** Percentage weight variation for Brand A and Brand B

Tablet No	Brand A % Wt. variation (Round Off)	Brand B % Wt. variation (Round Off)
1	-1.01	-1.27
2	-0.16	0.55
3	1.13	2.05
4	-0.85	2.01
5	-0.24	-1.24
6	-0.1	-0.86
7	-0.06	-0.45
8	0.91	0.88
9	2.79	-0.63
10	0.85	-1.8
11	-0.09	-0.78
12	-0.25	0.72
13	-0.26	-0.14
14	-1.1	-0.41
15	-0.61	0.15
16	0.12	0.5
17	-1.21	0.6
18	-0.62	0.63
19	0.05	0.23
20	0.52	-0.93

**Table 2:** Percentage weight variation for Brand A and Brand B

Tablet No	Brand A % Wt. variation	Brand B % Wt. variation
Highest	2.79	2.05
Lowest	1.21	1.27

Friability, drug assay, and disintegration test

The average percentage friability for Brand A was calculated to be 0.06 while the percentage friability for Brand B was calculated to be 0.01.

The drug assay test results show that the percentage of drugs available in Brand A is 94.7% and Brand B is 112.94%. As per the USP standards, 85%-115% is the acceptable range for the drug assay. Both drug brands A and B conform to the standards.

As per USP standards, the disintegration time for uncoated tablets is within 15 minutes. As per the QC tests performed, the disintegration time for Brand A is 6.69 minutes while the disintegration time for Brand B is 7.02 minutes. As per the results, both brands A and B conform to the USP standards [6-10].

The results are shown in Table 3.

**Table 3:** Friability, drug assay, and disintegration time of both brands

Brand Tablet	% Friability	% Drug	Disintegration Time (Min)
A	0.06	94.7	6.69
B	0.01	112.94	7.02

Dissolution test results

**Figure 3:**
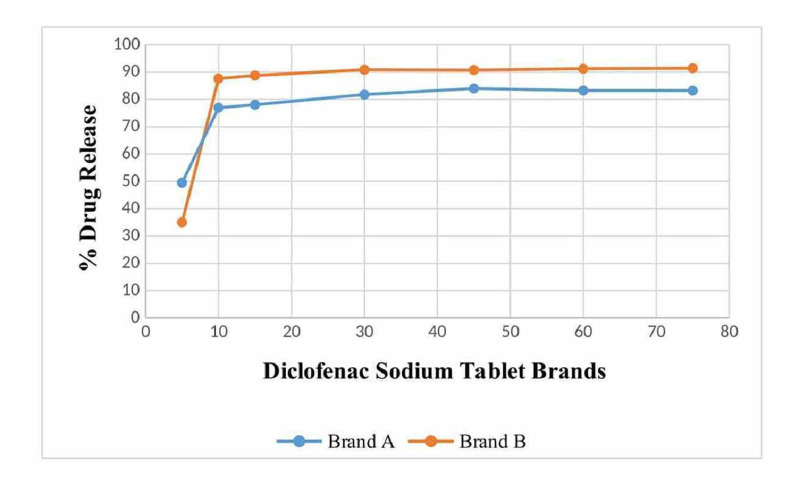
% dissolution for diclofenac sodium tablets (Brands A and B)

As per test results (Table 4), a Brand A tablet releases 83.003% drug in 45 minutes while a Brand B tablet releases 90.74% in the same time period. The variation in test results for Brand A can be due to random errors in measurement while using lab instruments or while using UV visible spectroscopy. The data provided indicated that both brands of diclofenac sodium were dissolved at a fast rate then, as shown in the graph, the percentage of drug released by Brand A increased during a shorter period of time than its peak rate of dissolution, whereby it continued slowly until almost all the drug was released and the same can be observed for Brand B as well. Both brands exhibited almost similar rates of release of the drug over a period of time (Figure 3).

**Table 4:** Drug dissolution for Brand A and Brand B

Sampling time/(min)	Brand A % Drug Dissolution	Brand B % Drug Dissolution
5	49.52	35.03
10	76.97	87.64
15	78.05	88.74
30	81.77	90.87
45	84.01	90.74
60	83.25	91.22
75	83.22	91.42

## Discussion

QC testing with diclofenac sodium tablets shows that Brands A and B conform as per the USP standards.

As per USP standards, a weight variation of 10% above or lower than the mean weight is allowed if the weight of the tablet is greater than 324 mg. Both Brands A and B tablets' weight variation limits were less than 3%, which is in the pharmacopeia limit, so both brands passed the weight variation test. Though both brands passed the weight variation test, the weight variation limit was higher in Brand A as compared to Brand B, which indicates that the flowability of powder blend B could be better than that of powder blend A because flowability has an impact on the powder filling process in the tablet die during compression. Another reason for the difference in weight variation in both brands could be the more glidant in the Brand B powder blend as compared to the Brand A powder blend.

As per the pharmacopeia, 1% loss of tablet during the transportation is permissible, and it was checked by friability equipment and the result shows that both brands of tablets have a loss of 0.06% and 0.01%, respectively, which means that both brands of tablets passed the friability test. Less loss of drug in tablet prediction by friability test in Brand B indicates that during the compression of the tablet, more force was applied for the production of Brand B tablets as compared to Brand A. Another reason could be that more amount of binder excipient was added to the Brand B tablet that provides more strength to the tablet and, ultimately, fewer chances of powder loss during transportation.

The disintegration time limit for uncoated tablets is 15 minutes and the result shows that the Brand A disintegration time was 6.69 minutes while it was 7.02 minutes for Brand B, which means that both disintegration times are under the pharmacopeia limit so both brands conform to the disintegration test. The drug assay revealed that Brand A tablets were having 94.7% drug while Brand B tablets were having 112.94% drug, and both amounts were under the pharmacopeia limit. The disintegration time provides an idea of when the tablet will disintegrate and reach dissolution. The tablet disintegration time of Brand A is less as compared to Brand B, which reflects that the powder blend of tablet A could have less binder excipient or there could be less force during tablet compression. The result of disintegration time also confirms the result of friability because the friability of Brand B is less, which means more strength and, ultimately, more disintegration time. 

The rate of drug release was confirmed by the dissolution tests of both brands (A and B) and it was found that in 75 minutes, almost 80% of the drug was released, and this was satisfactory from the dissolution point of view. The dissolution study was performed as a multiple point study to confirm the pharmacopeia monograph. The t-test was applied to the rate of drug release at different points for both Brands A and B. The degree of freedom (n-1) of the study was 6, as the total time interval was 7. The calculated value of both samples was 1.6786 and the tabulated t-value at 6 degrees of freedom is 2.447, which means that the calculated value is less than the tabulated value, which confirms the hypothesis. The accepted hypothesis confirms the reliability of the dissolution test.

## Conclusions

It can be deduced that both Brands A and B of diclofenac sodium tablets are within the pharmacopeia limit. The test performed on the two brands were: friability, weight variation, disintegration time, drug assay, and dissolution rate. Results related to QC testing of diclofenac sodium tablets show that such tests are necessary to determine the safety, efficacy, and bioavailability of a dosage form. A comprehensive range of analysis helps evaluate drugs qualitatively and quantitatively, and these tests must be performed from time to time in order to validate the drugs as per pharmacopeia standards and maintain drug safety and effectiveness for the human body.
